# New Detection Platform for Screening Bacteria in Liquid Samples

**DOI:** 10.3390/bios11050142

**Published:** 2021-05-01

**Authors:** Rita La Spina, Diana C. António, Radoslaw Bombera, Teresa Lettieri, Anne-Sophie Lequarré, Pascal Colpo, Andrea Valsesia

**Affiliations:** 1 European Commission, Joint Research Centre (JRC), Ispra, Italy; Rita.LA-SPINA@ec.europa.eu (R.L.S.); diana_conduto@hotmail.com (D.C.A.); radoslaw.bombera@gmail.com (R.B.); Teresa.LETTIERI@ec.europa.eu (T.L.); pascal.colpo@ec.europa.eu (P.C.); 2 European Commission, Joint Research Centre (JRC), Brussels, Belgium; Anne-Sophie.LEQUARRE@ec.europa.eu

**Keywords:** antimicrobial peptides, bacteria, water samples, detection, dark field, motility, fluorescence microscopy, quantum dots

## Abstract

The development of sensitive methods for the determination of potential bacterial contamination is of upmost importance for environmental monitoring and food safety. In this study, we present a new method combining a fast pre-enrichment step using a microporous cryogel and a detection and identification step using antimicrobial peptides (AMPs) and labelled antibodies, respectively. The experimental method consists of: (i) the capture of large amounts of bacteria from liquid samples by using a highly porous and functionalized cryogel; (ii) the detection and categorisation of Gram-positive and Gram-negative bacteria by determining their affinities toward a small set of AMPs; and (iii) the identification of the bacterial strain by using labelled detection antibodies. As proof of concept, the assessment of the three steps of the analysis was performed by using *Escherichia coli* and *Bacillus sp.* as models for Gram-negative and Gram-positive bacteria, respectively. The use of AMPs with broad specificity combined with labelled antibodies enabled the detection and potential categorization of a large spectrum of unknown or unexpected bacteria.

## 1. Introduction

The development of methods for the rapid detection and identification of bacteria is of upmost importance for environmental monitoring and food safety [1]. Standardized methods of detecting bacteria in water and other matrices are based on microbial plating and enumeration [2,3]. These methods are highly accurate and sensitive but require at least 24 h to obtain the results of analysis [2]. Other standardized methods [4] for detecting bacteria are based on testing the genetic and immunological features of microorganisms. Polymerase chain reaction (PCR) is a method that targets known gene sequences and thus offers a highly specific analysis of a wide range of bacteria. Quantitative PCR (qPCR) analyses [5] can be advantageously used for increasing the sensitivity up to single-cell level. However, qPCR-based assays are time-consuming and require specific primers for a subset of bacteria, reducing the detection spectrum. Enzyme-linked immunosorbent assay (ELISA), which relies on very specific antibodies for detecting bacteria, is widely used but also requires pre-enrichment of the bacteria samples [6]. The pre-enrichment phases are time consuming and can vary between 24 and 48 h according to the sample nature [7]. Recently, alternative methods were developed to provide rapid analysis responses without a significant decrease in sensitivity. For instance, lateral flow assay (LFA) is a rapid test format using antibodies as bioreceptors and colorimetry as detection method. Other methods use specific antibody-coated magnetic beads to separate target bacteria from the sample matrix and their recognition is subsequently amplified by antibody-functionalized nanoparticles and quantum dots [8,9]. Magnetic nanoparticles [10] and gold nanoparticles [11] bearing antibodies are used for the separation of *E. coli* from the complex matrix and then analyzed by colorimetric assay or Raman spectroscopy. Flow cytometric methods are also widely used for measuring bacteria in water [12].

In this work, we present a new method combining a pre-enrichment step using a microporous cryogel and a detection step using antimicrobial peptides (AMPs) as bioreceptors and labelled antibodies for identification. The use of highly porous cryogel for bacteria pre-concentration was already successfully investigated, showing a high capacity for harvesting [13]. In particular, pHEMA-AEM (2-hydroxyethylmethacrylate (HEMA) 2-Aminoethyl methacrylate hydrochloride (AEM)) is a polymeric cryogel synthesized through a cryostructuration method characterized by a large inner surface area, resulting in a high capacity for bacteria harvesting, even from complex samples [14]. Antimicrobial peptides (AMPs) bioreceptors were shown to be an interesting alternative to antibodies due to their broad recognition spectrum [15,16,17]. AMPs are a subset of peptides presenting strong bactericidal activity against Gram-positive and Gram-negative bacteria according to specific patterns [18]. The binding of cationic AMPs to the bacterial surface is driven by electrostatic interactions with the negatively charged cell wall components of Gram-positive or the lipopolysaccharides of Gram-negative bacteria [19]. Literature shows that a set of AMPs with overlapping but non-identical specificities to different microbial targets enables detection and categorization of unknown bacteria, proteins, toxins, and viruses [17,20]. The sensing platform presented in this work consists of a silicon chip coated with poly(ethylene) oxide (PEO) thin film which has the interesting property of being cell-repellent while allowing protein printing under specific conditions [21,22]. The developed assay consists of three steps: (i) entrapment of bacteria in the cryogel; (ii) desorption of bacteria from the cryogel and measurement of their affinities toward immobilized AMPs; and (iii) bacteria identification using specific labelled antibodies. The three assay phases were assessed for the detection of *E. coli* K12 as Gram-negative (Gram (−)) and Bacillus sp. 9727 Gram-positive (Gram (+)) bacteria using cecropin B and cecropin P1 AMPs.

## 2. Materials and Methods

### 2.1. Chemicals

2-hydroxyethylmethacrylate (HEMA), 2-Aminoethyl methacrylate hydrochloride (AEM) *N,N′*-methylenebisacrylamide (MBAA), ammonium persulphate (APS), 1,2-bis(dimethylamino)ethane (TEMED) and sodium chloride, and cecropin B and cecropin P1 were purchased from Merck KGaA (Darmstadt, Germany). Phosphate buffer saline (PBS) was purchased from Gibco Italia. Peptone, meat extract, tryptone, yeast extract and Noble Agar were purchased from BD Diagnostics (Franklin Lakes, NJ, USA). Antibodies against *E. coli* (Polyclonal anti-*E. coli* ab13627) were purchased from Abcam (Cambridge, UK). Quantum dots (QDs) and Qdot™ Incubation Buffer were purchased from Invitrogen (code Q10101 MP and Q20001 MP, respectively).

### 2.2. Bacterial Cultures

Bacterial strain *E. coli* K12 (DSM No: 6897) was purchased from the American Type Culture Collection and *Bacillus sp.* 9727 (DSM No: 9727) strains were purchased from Leibniz Institute DSMZ–German Collection of Microorganisms and Cell Cultures. All strains were maintained in rich media and kept at −20 °C for long storage. The *E. coli* strain was cultured in Luria–Bertani medium (LB) at 35 °C and *Bacillus sp.* was cultured in alkaline nutrient broth (AN) at 30 °C, with agitation, according to supplier recommendations. Agarified LB and AN plates were prepared, with 15 g L^−1^ of Noble Agar, for colony-forming unit (CFU) enumeration. To avoid unspecific interaction with media components, freshly grown cells were washed in PBS before analysis.

### 2.3. Synthesis and Physico-Chemical Characterization of the P(HEMA-AEM) Cryogels

The P(HEMA-AEM) cryogel was obtained by mixing 2 mmol of AEM, 3.9 mmol of HEMA and 2 mmol of MBAA in 9 mL of water. After degassing the solution for 30 min, 1% *w*/*w* APS/TEMED of the total monomers was added to the solution. Each cryogel was synthesized in 0.5 mL solution in a glass tube of 7 mm diameter and frozen for 24 h at −12 °C. The cryogel was then washed with an increasing percentage of water and ethanol (0, 30, 50, 70, 90, 100%) and left to dry at room temperature (RT). The cryogel has an approximate dimension of a few cubic mm.

### 2.4. Surface Characterization

The morphology of the cryogel was examined by scanning electron microscopy (SEM, Nova 600i Nanolab, Thermofisher, The Netherlands). Prior to analysis, the cryogel was washed with an increasing percentage of ethanol and then treated using critical point drying (CPD) to substitute ethanol with CO_2_ molecules.

### 2.5. Adsorption of Bacteria onto P(HEMA-AEM) Cryogels in PBS and Spiked Water Samples

Adsorption experiments were carried out with the cryogel immersed in buffer at RT with gentle shaking. Aliquots of 45 mg of dried P(HEMA-AEM) cryogel were added to 4 mL of PBS containing *E. coli* bacteria at different concentrations ranging from 10^4^ to 10^8^ cells mL^−1^ and *Bacillus sp.* at 10^8^ cells mL^−1^. In addition, the same series of experiments was carried out by adding the cryogel ranging from 10 to 90 mg to 8 mL of bacteria at 10^8^ cells mL^−1^. The bacteria adsorption to P(HEMA-AEM) cryogel was also performed with *Bacillus sp.* spiked buffer at 10^8^ cells mL^−1^. Experiments on adsorption kinetics were performed to determine the required time of incubation to reach the equilibrium of adsorption for a known concentration of bacteria. Specifically, these experiments were carried out at RT by quantifying the amount of *E. coli* bacteria adsorbed by the P(HEMA-AEM) cryogel as a function of the time up to 24 h. In addition, the adsorption of bacteria in different matrices was evaluated by spiking environmental water (Lago Maggiore, Italy) and bottled mineral water with *E. coli* at known concentrations (10^4^, 10^6^ or 10^8^ cells mL^−1^), according to French Norm T3 [23]. All experiments were performed in triplicates.

### 2.6. Quantification of Adsorbed Bacteria onto P(HEMA-AEM)

The amount of *E. coli* adsorbed onto P(HEMA-AEM) was determined by subtracting the initial bacteria concentration with the bacteria concentration remaining in supernatant solution at different time points measured by CFU analysis. The adsorption of *E. coli* at the initial concentration of 10^7^, 10^8^ cells mL^−1^ was also quantified by turbidimetric analysis assuming a correspondence of 10^9^ cells mL^−1^ at optical density at 600 nm (OD_600nm_) of 1 absorbance unit (AU). As control, the initial concentration of bacteria was analyzed during the time of incubation. The adsorption was measured by spectrophotometry at 600 nm (Biowave II WPA lab vision, Cambridge, UK).

The adsorption capacity of the cryogel is calculated as % of adsorbed bacteria, following this equation:

(1) $$A=\frac{C_{e}-C_{0}}{C_{o}}\times 100,$$

where *C*_0_ is the initial concentration of viable *E. coli* bacteria (cells mL^−1^) in solution, and *C_e_* is the bacteria concentration remaining in supernatant solution at the equilibrium.

### 2.7. Quantification of Adsorbed Bacteria onto P(HEMA-AEM) in Flow Condition

The adsorption of bacteria was determined in flow condition at RT, in PBS, at flow rates of 0.05, 0.1, or 0.3 mL min^−1^. An amount of 30 mL of bacteria was prepared at the initial concentration of 10^8^ cells mL^−1^. The concentration of bacteria contained in the supernatant was determined by performing turbidimetric analysis of fractions of 800 µL collected at different time points. The percentage of bound bacteria was then calculated by dividing the initial bacteria concentration subtracted with the concentration remaining in supernatant solution by the initial concentration of bacteria.

### 2.8. Elution of Adsorbed Bacteria from P(HEMA-AEM) Cryogel

An amount of 45 mg of P(HEMA-AEM) cryogel was incubated for 6 h in 10 mL of *E. coli* and *Bacillus sp.*, at the concentration of ~4 × 10^7^ cells mL^−1^. Then, the cryogels were transferred in vials containing 4 mL of 1 M NaCl and phosphate buffer at pH 12 to detach *E. coli* and *Bacillus sp.* from the cryogel, respectively. The concentration of the eluted bacteria was measured by turbidimetric and CFU analysis.

### 2.9. Bacterial Motility Measurements

A silicon wafer was coated with a 100 nm thick layer of PEO. For the PEO deposition, pure diethylene glycol dimethyl ether (DEGDME, (CH_3_OCH_2_CH_2_)_2_O) (Sigma Aldrich, Milan, Italy) used as precursors is heated at 45 °C. Plasma polymerization was carried out in continuous plasma discharge mode with a power of 20 W. The PEO layer was functionalized by incubating the surface with 50 µg mL^−1^ AMPs solution, and rinsed with milliQ water. Sealable microchannels (Ibidi Germany, μ-slide VI ^0.4^, volume of the channel: 30 μL, growth area: 60 mm^2^) were then used to transfer the liquid samples to the functionalized PEO layer. Optimization and confirmation of the surface functionalization with AMPs bioreceptors was performed by surface plasmon resonance (SPR Biacore T200, GE Healthcare Life Sciences). The channel was filled with 30 μL of bacteria suspension at different concentrations. After 30 min of incubation, almost 80% of the cells were settled and therefore interacted with the functionalized PEO layer. Before rinsing the channel, a video of the settled bacteria was acquired for a total of 160 frames at a rate of 1 frame per second. The video was analyzed using ImageJ for determining the vectorial (X/Y) displacement of each cell versus time. The average displacement of the bacteria population was calculated, with the displacement being proportional to the motility. The motility of each strain interacting with each PEO-AMPs surface was compared with the motility of the same strain interacting with the non-AMPs functionalized PEO surfaces. Images and video acquisition were obtained in dark field (DF) microscopy (Zeiss, Axio Imager 2). After motility analysis, channels were rinsed with 1 mL PBS. The remaining bacteria on the surface were then subjected to the bacteria identification step, as described below.

### 2.10. Bacteria Identification Test with Labeled Antibodies

The identification of bacteria immobilized on the sensing platform was performed. As proof-of-concept, the test was only performed for the detection of *E. coli* bacteria. Anti-*E. coli*-QDs conjugates were prepared as follows: 10 µL of anti-*E. coli* (0.4 mg mL^−1^) in Qdot™ Incubation Buffer were incubated with 200 µL of QDs (0.1 µM) for 30 min and gently stirred [9]. After rinsing the sensing platform surface, cells attached to the functionalized surface were incubated for 60 min with the anti-*E. coli*-QDs complex and rinsed before analysis. The comparison of the images obtained by DF with those obtained by fluorescence exposure allowed the identification of the *E. coli* cells and the estimation of the identification efficiency. *E. coli* bacteria and their identification by anti-*E. coli*-QDs complex were also tested using mineral water instead of PBS. The image acquisition was obtained in dark field (DF) and fluorescent microscopy (FM) (Zeiss, Axio Imager 2).

## 3. Results and Discussion

**Figure 1:**
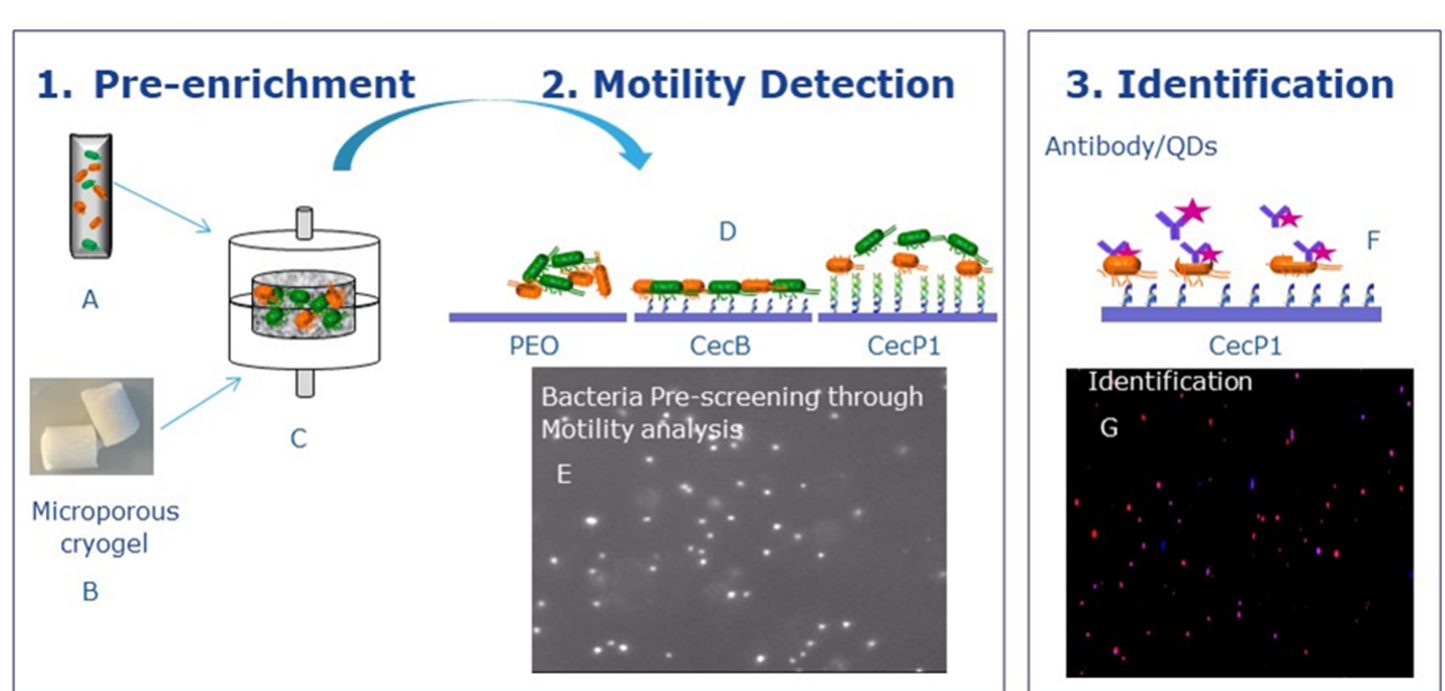
Schematic representation of the method implemented for the pre-enrichment, detection, and identification of bacteria.

Figure 1 shows a schematic representation of the experimental method developed for the screening, detection, and identification of bacteria in water samples.

In the pre-enrichment phase (step1, Figure 1A) the sample containing *E. coli* and *Bacillus sp*. (represented by orange and green ellipsoids, respectively) was incubated with the cryogel (Figure 1B) for bacteria entrapment (Figure 1C).

The bacteria were then desorbed from the cryogel and transferred to the sensing platform. The proposed sensing platform (Figure 1D) consisted of a silicon surface coated with a poly(ethylene) oxide (PEO) thin film, functionalized with CecB and CecP1 peptides (blue and green, respectively). Desorbed bacteria were flowed over the surface and incubated for 30 min settling time. DF microscope was focused on the surface of the chip in order to detect the settled bacteria (Figure 1E) and evaluate their motility. The bacteria were screened and classified by comparing their motility on different AMPs-coated surfaces to their motility on the PEO surface, assuming the latter as their natural motility due to their weak interaction with PEO. High (respectively low)-affinity of bacteria toward the AMPs is characterized by a low (respectively high) motility. Next, the identification step was carried out by using secondary antibodies conjugated with QDs (Figure 1F,G). The latter act as a probe to identify the bacteria type using fluorescent microscopy. The use of specific antibodies enables the detection of known and expected bacteria. Furthermore, the superimposition of the DF and fluorescence microscopy images enabled the detection of bacteria not recognized by the specific antibody but immobilized on the sensing surface due to the broad recognition spectrum of the AMPs.

### 3.1. Physico-Chemical Characterization of P(HEMA-AEM) Cryogel

A P(HEMA-AEM) polymeric 3D structure was synthesised through a cryostructuration method. At temperature below the solvent melting point, the polymerisation occurred around the frozen crystal of solvent, forming a stable three-dimensional network. Thawing of the frozen solvent produced cavities in the order of micron and generated micropores that replicated the crystals. The first part of the work demonstrated the bacteria adsorption capacity of the P(HEMA-AEM) cryogel.

#### 3.1.1. Adsorption of Bacteria in PBS and Spiked Water Samples

Binding studies under gentle shaking conditions were performed to assess the adsorption capacity of the P(HEMA-AEM) cryogel towards *E. coli* and *Bacillus sp.* Figure S1 shows a scanning electron microscopy image of the P(HEMA-AEM) cryogel before and after incubation with bacteria showing its 3D microporous structure and clearly evidencing the binding of bacteria after 6 h of incubation. Kinetic studies of adsorption of bacteria onto cryogel were performed in PBS at *E. coli* concentrations of 10^4^, 10^7^, and 10^8^ cells mL^−1^. The ratio between the cryogel and the bacteria suspension was 45 mg of dried cryogel in 4 mL of PBS bacteria suspension. The adsorption kinetics curves of *E. coli* are shown in Figure S2. Comparative studies of *E. coli* adsorption onto P(HEMA-AEM) cryogel in the range of studied concentrations suggest the system reaches the saturation point after 52 ± 13, 98 ± 7, and 184 ± 18 min with *E. coli* concentrations of 10^4^, 10^7^, and 10^8^ cells mL^−1^, respectively. In addition, the results show that for 45 mg of dry cryogel in 4 mL of *E. coli* bacteria at 5.7 × 10^4^, 5.8 × 10^7^, and 6.1 × 10^8^ cells mL^−1^ are able to adsorb 5.69 × 10^4^, 5.7 × 10^7^, and 5.4 × 10^8^ cells mL^−1^, which corresponds to 99%, 98%, and 89% of the initial concentration of bacteria, respectively.

The effect of the matrix (PBS, mineral water and lake water) on the cryogel adsorption capacity was then studied for different bacteria concentration i.e., 10^4^, 10^6^, and 10^8^ cells mL^−1^, respectively, using the same condition of 45 mg of dried cryogel in 4 mL of PBS bacterial suspension (Figure S3). A comparison of the experimental measurements after 24 h incubation for different concentrations of bacteria shows that there is no significant effect of the matrix on the binding capacity of the cryogel, even at a lower initial number of bacteria.

Binding studies of bacteria onto the cryogel were carried out by keeping the amount of dried cryogel constant towards the volume of *E. coli* bacteria (45 mg: 4 mL of bacteria in PBS) and instead varying the concentration of *E. coli* bacteria from 10^4^ to 10^8^ cells mL^−1^ (Figure S4). The same trend was obtained from the incubation of the P(HEMA-AEM) cryogel with the *Bacillus sp.* These experiments show that the cryogel surface adsorbs both Gram (+) and Gram (−) bacteria with the same affinity.

**Figure 2:**
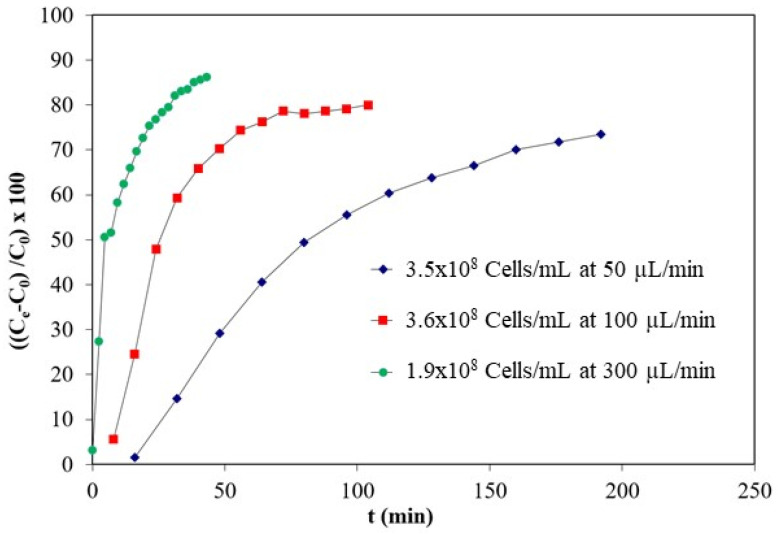
Breakthrough curves of *E. coli* bacteria in PBS at similar inlet concentrations and temperatures but different flow rates at 50, 100, and 300 µL min^−1^. The mass of the cryogel was 30 mg.

In order to reduce the time necessary to reach the adsorption saturation, further experiments in flow conditions were performed at RT, in PBS, at the bacteria concentration of 3 × 10^8^ cell mL^−1^ and at the flow rates of 0.05, 0.1, or 0.3 mL min^−1^. The dried mass of the cryogel was 30 mg. Fractions of 800 µL were collected at different time points and further analyzed by turbidimetric method. Breakthrough curves of *E. coli* bacteria in PBS are presented in Figure 2 showing that increasing the flow rate sharpened the breakthrough curve enabling the binding saturation in shorter time. The results show that for an initial concentration in the range of 10^8^ cells mL^−1^ and a flow rate of 300 µL min^−1^, more than 80% of the bacteria are entrapped in the cryogel after 20 min (versus several hours in static incubation). The time needed for the pre-enrichment can be optimized by adapting the mass of the cryogel, and adjusting the flow-rate to the volume of sample to be analyzed.

#### 3.1.2. Elution of Adsorbed Bacteria from P(HEMA-AEM) Cryogel

The procedure for bacteria desorption from the cryogel was optimized for both bacteria strains. Both *E. coli* and *Bacillus. sp.* bacteria at a concentration of 4 × 10^7^ cell mL^−1^ were incubated for 6 h and the adsorption of bacteria was in the order of 90%.

In this case, 45 mg of cryogel were equilibrated in 10 mL of bacterial suspension. For the desorption, the same cryogels were suspended in a water solution containing 1 M NaCl, which enabled the release of 45% of the *E. coli* bacterial cells attached on the cryogel within 1 h (data not shown). The bacterial release is favored due to screening of the electrostatic forces that drive the bacteria binding to the cryogel by the ions contained in the salt solution. The same solution was less effective in releasing only 12% of the adsorbed *Bacillus sp*. resulting most likely from a different binding mechanism. The use of 0.01 M phosphate buffer at pH 12 was more effective; enabling the release of 45% of *Bacillus sp.* from the cryogel. After elution, the bacteria dispersion was centrifuged and dispersed in PBS.

To summarize, these results show that the positively charged P(HEMA-AEM) is a material of choice for pre-enrichment steps considering that most of the Gram (+) and Gram (−) bacteria have a negatively charged membrane. In addition, its capability to entrap and elute large amounts of bacteria (98% for an initial concentration of 10^8^ cells mL^−1^ in spiked PBS and environmental water, 50% of controlled elution efficiency) makes the cryogel very efficient in harvesting pathogens, which are often present at low concentrations in environmental samples, making their analysis difficult. To improve the speed of harvesting, the bacteria capture can also be advantageously carried out in flow conditions by adapting the cryogel to a solid phase extraction (SPE) disk holder, for bacteria harvesting directly in situ during the sampling campaign, for instance. The concentration and release of bacteria in solution would benefit a number of techniques for detection of bacteria, which require pre-enrichment steps.

### 3.2. Detection and Identification of Bacteria

#### 3.2.1. Bioreceptor Immobilization

The immobilization of the AMPs bioreceptors on the PEO surface was first assessed using the surface plasmon resonance (SPR) technique. The SPR technique enables a real-time monitoring of the peptide immobilization process. SPR chips were coated with a 30 nm layer of PEO. The cecropin B (CecB) and cecropin P1 (CecP1) AMPs solutions were flowed onto the PEO-coated chip in different buffers in order to determine the optimal loading conditions (data not shown). The resulting SPR responses confirmed the correct surface functionalization with the two AMPs and show that the surface loading was different for the two peptides (Figure S5). AMPs have short sequences characterized by positively charged, negatively charged, and hydrophobic domains, which determine the charge distribution in water at given pH. Optimal immobilization of Cec P1 and Cec B was achieved in glycine buffer at pH 11. pH 11 being very close to their pKa, the two AMPs are in a neutrally charged state in this condition. Although PEO is slightly negatively charged at pH 11, the attachment of the AMPs to the PEO surface is favoured by the absence of electrostatic repulsion. The successful AMPs attachment to the PEO is characterized (Figure S5) by an increase in the RU signal of about 10,000 RU and 6000 RU for the CecB and CecP1, respectively, corresponding approximately to a surface density of 10 pg mm^−2^ and 6 pg mm^−2^. After glycine rinsing, some loosely bound peptides were removed from the surface, resulting in a decrease in the SPR signals (Figure S5). The signals resulting from the number of peptides stably bound on the PEO surface was thus 7500 RU and 2700 RU, corresponding to 7.5 pg mm^−2^ and 2.7 pg mm^−2^, respectively, for the CecB and the CecP1. These values correspond approximately to a complete monolayer of AMPs covering the PEO surface.

#### 3.2.2. Determination of Bacteria Affinity toward AMPs by Motility Measurement

First, the affinity of the bacteria towards the different AMPs was assessed by monitoring their motility close to the surface using dark field microscopy image analysis (Figure 1). Literature shows that PEO-like surfaces have low adhesive properties and present highly repulsive forces toward bacterial membranes [24,25]. In this study, we assume that the natural motility of bacteria on the PEO surface is not reduced as a result of their weak interactions with this surface. When PEO film is functionalized with AMPs, the bacteria approaching the surface interact with the AMPs through their membranes. If the AMPs have a high affinity for the bacterial membranes, the bacteria bind to the AMP-functionalized surface, resulting in the hindering of their motion. The inhibition of the motility is thus directly related to the affinity of the AMPs to the bacterial membrane. The displacement of single bacteria versus time was monitored by dark field microscopy (DF). The total displacement, i.e., motility of a bacterium within a given image time frame (in the orders of few minutes), was measured. Using 10× magnification optics, the displacement of hundreds of bacteria was monitored simultaneously. The average displacement of the bacteria population was then calculated as the average of the displacements of each bacterium measured individually. The single bacterium in DF was characterized by a number of bright pixels within a dark background. The bacteria are characterized by typically spherical or elliptical shapes. By considering its geometrical center, the bacterium was identified by a square-shaped object consisting of bright pixels, positioned around the geometrical center (Figure S6a). The image analysis software calculated the variations of the positions of the groups of bright pixels of each bacterium. This calculation was done for each frame of the acquired sequences. The sum of the position variations of the bacterium during the observation time represents the total displacement of a bacterium. Examples of displacements measured on PEO surface by this method are shown in Figure S6b,c.

#### 3.2.3. Bacterial Motility Measurements

Samples with known bacterial concentration were incubated for 30 min on non-functionalized PEO and AMPs functionalized PEO to enable the settlement of a large number of bacteria towards the surfaces. Then, bacterial motility was calculated by averaging of the measured displacement of the different bacteria as explained in a previous paragraph.

**Figure 3:**
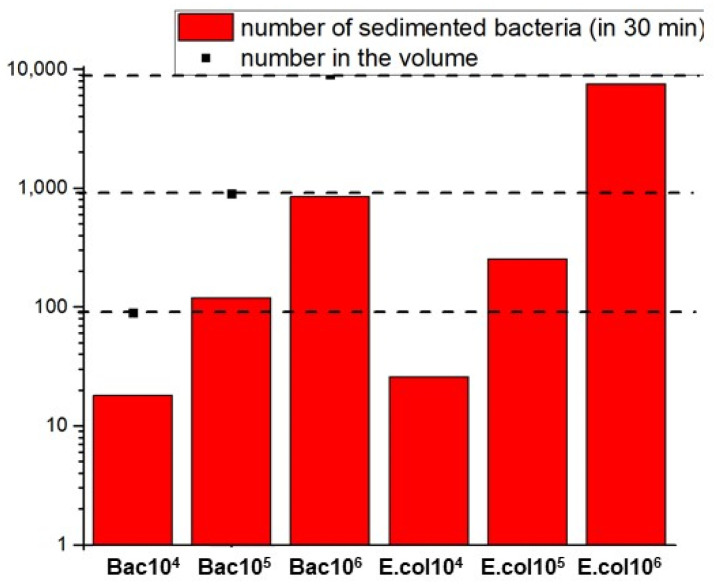
Total number of settled bacteria cells, *E. coli* and *Bacillus sp.*, as a function of the initial concentration. The dashed line represents the initial number of bacteria present in the channel volume.

Figure 3 presents the number of settled bacteria during the motility analysis as a function of the initial concentration and the number of bacteria contained in the channel volume. For a field of view of 0.16 mm^2^, concentration of 10^4^ cells mL^−1^ and a settlement time of 30 min, around 20 bacteria among the initial 300 bacteria present in the volume reached the surface. As reference, 5 bacteria has been indicated as a sufficient number to make a reliable quantitative detection [26].

**Figure 4:**
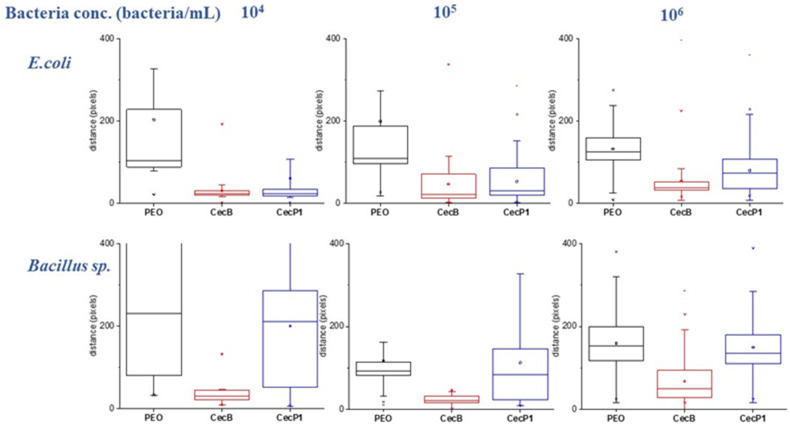
Motility analysis of *E. coli* and *Bacillus sp.* on PEO, cecropin B (CecB), and cecropin P1 (CecP1) at different concentrations of bacteria.

**Figure 5:**
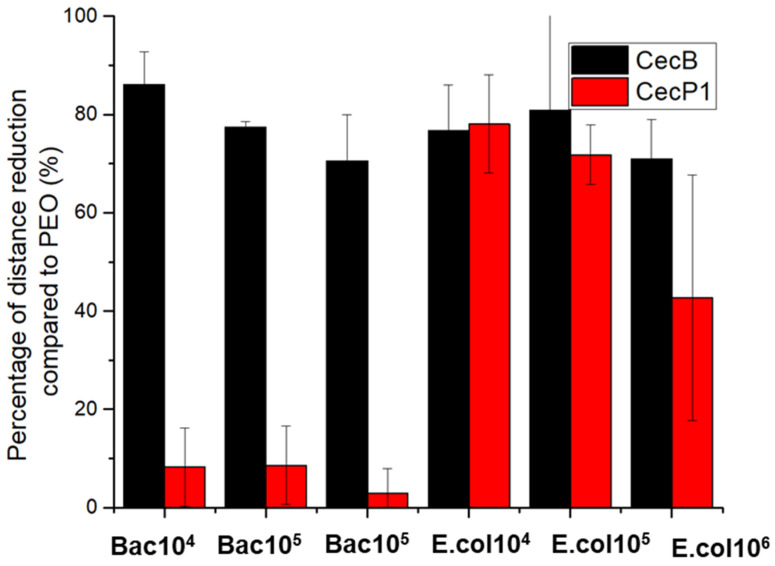
Summary of the motility analysis of *E. coli* and *Bacillus sp.* on PEO, cecropin B (CecB), and cecropin P1 (CecP1) at different concentrations of bacteria. It shows the percentage of reduction in motility compared with the motility of bacteria on PEO.

Figure 4 shows the motility measurements on the bare PEO and AMPs functionalized PEO (average distance travelled by the counted bacteria) for different bacteria concentrations of *E. coli* and *Bacillus sp*. For both bacteria strains, the average distance travelled was independent from the concentration, with a decrease in the standard deviation occurring as the concentration increased, as a result of a better statistical distribution. A summary of the motility results is shown in Figure 5.

Both CecB and CecP1 induced a large reduction in the *E. coli* motility for all concentrations. The average distance travelled by *E. coli* was reduced by about 80% and 60% for CecB and CecP1, respectively, to those measured on bare PEO. This motility reduction showed that both AMPs interact strongly with the *E. coli* bacteria membranes that have a slightly higher affinity for the CecB.

In addition, *Bacillus sp.* showed high-affinity with CecB, as a result of a reduction in the average travelled distance of about 80% while showing a lower affinity with CecP1 since the travelled distance was very similar to the one measured on PEO (motility reduction lower than 15%). The obtained results are in agreement with the literature [27] and show that CecB AMPs have a high-affinity for both Gram (+) and Gram (−) bacteria, while the CecP1 only shows a high-affinity for the Gram (−) bacteria.

Motility of bacteria desorbed from the cryogel was measured. The graph in Figure 3 was used to determine the concentration of bacteria desorbed from the cryogel by using the number of settled bacteria. The parameters of desorption were 1 M NaCl for *E.coli* and 0.01 M phosphate buffer at pH 12 for *Bacillus sp.* The amount of *E.coli* bacteria detected by the surface was in the order of 1650 bacteria, which corresponds to an *E.coli* concentration between 10^5^–10^6^ cells/mL. Moreover, the amount of *Bacillus sp.* bacteria detected by the surface is in the order of 850 bacteria, which corresponds to a *Bacillus sp.* concentration below ~10^6^ cells/mL.

The results of Figure S7 show that the motility reduction, i.e., bacteria affinity towards AMPs, is not altered by the solvent used for the desorption step. *E.coli* bacteria present similar and high-affinity to both AMPs, whereas *Bacillus sp.* showed high- and low-affinity with CecB and CecP1, respectively. The affinity measurements suggest that by using a small set of AMPs, a pattern of recognition could be established, i.e., bacteria having high-affinity to CecB and CecP1 could most likely be Gram (−) bacteria, and bacteria having high- and low-affinity with CecB and CecP1, respectively, are most likely Gram (+) bacteria. It is clear that this concept must be validated and expanded by using other bacterial strains and AMPs set.

In the current experimental setup, the bacterial motility detection in the proximity of the functionalized surfaces requires the sedimentation of the bacteria. This is important to improve the statistical analysis of their motility, even though it would be sufficient to follow the motility of a single bacterium.

### 3.3. Bacteria Identification by Labelled Antibodies

In the previous section we demonstrated that bacterial motility can be used to determine the affinity of the bacteria towards the AMPs, giving information on the family of bacteria present in the samples, which could be unknown in, e.g., environmental samples.

To validate the results of the motility assay, the identification of the bacterial species immobilized on cecropin B AMPs was performed using *E. coli* antibodies conjugated with quantum dots. To co-localize the bacteria immobilized on the surface and bacteria specifically bound by the antibodies we performed the following two-step experiment. First, an area of the chip was scanned by DF microscopy. The detected bacteria are colored in blue by the image analysis software. After exposure to the fluorescent antibodies, the same area is scanned in fluorescence microscopy mode (FM). In this case, bright objects are colored in red by the software. When superposing the two images, the appearance of pink objects indicates the co-localization of the bacteria detected by DF and the bacteria bound by the antibodies.

**Figure 6:**
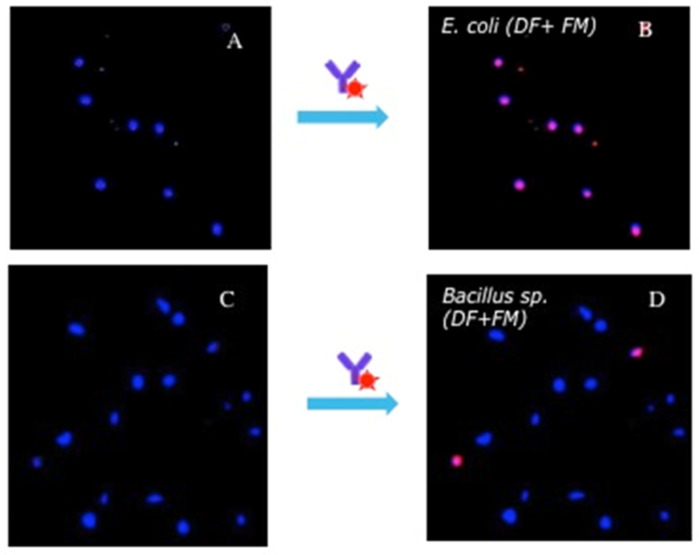
(**A**) DF image of *E. coli* bacteria immobilized on cecropin B AMPs. (**B**) Overlapping of DF and fluorescent images of the same area (fluorescent bacteria in pink). (**C**) DF image of *Bacillus sp*. bacteria immobilized on cecropin B AMPs (bacteria in blue). (**D**) Overlapping of DF and fluorescent images in the same area (fluorescent bacteria in pink).

In the first test, the Anti-*E. coli*-QDs were flowed over a surface previously incubated with *E. coli* and *Bacillus sp*. separately (Figure 6).

The blue spots in Figure 6A,C show bacteria bound to the CecB functionalized surfaces. Figure 6B shows that all bacteria immobilized are bound by the specific Anti-*E.coli* antibodies. On the other hand, Figure 6D shows that the large majority of bacteria are not recognized by the Anti-*E.coli* antibodies. Only two bacteria, over more than twenty, are colored in pink, most likely due to the nonspecific adsorption of the Anti-*E.coli* onto the *Bacillus sp*.

**Figure 7:**
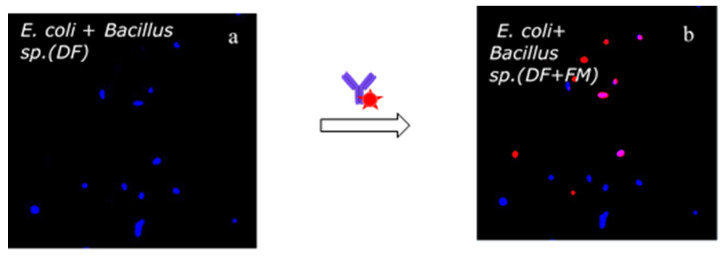
(**a**) DF image of *E. coli* and *Bacillus sp.* bacteria immobilized on cecropin B AMPs (bacteria in blue). (**b**) Overlapping of DF and fluorescent images of the same area (fluorescent bacteria in pink).

Further experiments were carried out by mixing *E. coli* and *Bacillus sp*. in mineral water and flowing the sample over the same CecB functionalized surface. The DF scan showed the immobilized bacteria without giving information about *E. coli* and *Bacillus sp.* (Figure 7a). The superimposed DF and FM images of Figure 7b enabled identification of the presence of *E. coli* on the surface due to the fluorescent pink dots, and at the same time to the presence (blue dots) of different bacteria strains than *E. coli* bacteria (in the present case, *Bacillus. Sp*.). Note that the red dots present in Figure 7 are most likely resulting from *E. coli* bacteria not present in the initial DF images that moved on due to the flow shear stress.

## 4. Conclusions

The results show that P(HEMA-AEM) microporous cryogel is a material of choice to enrich bacteria concentration from different samples and matrices. The time needed for the pre-enrichment is controlled by optimizing the mass of the cryogel and the flow-rate to the volume of sample to be analyzed. Captured bacteria are then eluted from the cryogel straightforwardly to detect them on a chip by DF microscopy and image analysis. As proof of concept, this study shows that motility monitoring, i.e., affinity towards a small set of AMPs, allows us to differentiate bacteria families by looking at *Escherichia coli* and *Bacillus sp.* as models for Gram-negative and Gram-positive bacteria, respectively. The screening potential of our system can be improved by analyzing bacterial motility patterns by using a larger set of AMPs with complementary affinity [15].

The advantages of this approach compared with direct identification through the antibodies consist of: (i) the use of a label-free method for detecting the presence of possible pathogenic bacteria in water samples; (ii) fast response in detecting the bacteria since the screening is carried out in 30 min; and (iii) the use of dark field and optical microscopy as detection methods, which is often available as laboratory equipment and also does not need specialized personnel.

In addition, bacteria immobilized on the AMPs can be specifically recognized by incubating the immobilized bacteria with the specific fluorescently labelled antibodies (e.g., Anti-*E. coli*-QDs complexes). Then, superimposition of DF and FM images enables us to distinguish between bacteria recognized by antibodies and bacteria not recognized, i.e., to determine whether other families of bacteria are present in the sample but are not recognized by the specific antibodies. Further miniaturization using a smartphone-like device of DF microscopy could also be implemented for fast and in the field measurements [28,29]. The ability of the biosensor to detect targeted pathogens in low concentrations among several other bacterial species and cells needs to be further addressed.

## Data Availability

The datasets generated during and/or analysed during the current study are available from the corresponding author on reasonable request.

## Supplementary Materials

The following are available online at https://www.mdpi.com/article/10.3390/bios11050142/s1, Figure S1: Scanning electron microscopy image of (**a**) microporous cryogel and (**b**) *E. coli* bacteria adsorbed on the P(HEMA-AEM) cryogel. Figure S2: Adsorption kinetics of *E. coli* bacteria (initial concentration = 10^4^, 10^7^, and 10^8^ cells mL^−1^) onto P(HEMA-AEM) cryogel in PBS. Figure S3: Comparative adsorption of *E. coli* bacteria at the concentration of 10^4^, 10^6^, and 10^8^ cells mL^−1^ in PBS, in lake water and in commercial mineral water. Figure S4: Adsorption of bacteria ranging from 10^3^ to 10^8^ cells mL^−1^ *E. coli* and *Bacillus sp*. bacteria. Figure S5: PEO surface functionalization with cecropin P1 (CecP1) and cecropin B (CecB) monitored by SPR. Figure S6: Cell motility is presented as a shifting of cell position regarding the geometrical center at time zero, travelled vectorial distance of the cell along the 160 frames, and variation of the X and Y position regarding the time zero (starting position). Figure S7: Motility analysis of (**a**) *E. coli* and (**b**) *Bacillus sp.* desorbed from the cryogel on PEO, cecropin B (CecB) and cecropin P1 (CecP1).
