# Supplementary material for: New Detection Platform for Screening Bacteria in Liquid Samples
Source: Biosensors (Basel). 2021 May 1;11(5):142. doi: 10.3390/bios11050142 (PMC8147366; doi:10.3390/bios11050142)
Supplement: Supplementary file 1 [file biosensors-11-00142-s001.zip › biosensors-1167650-supplementary.pdf]

biosensors

Supporting Information

New Detection Platform for Screening Bacteria in Liquid Samples

Rita La Spina [1,†], Diana C. António [1,†,‡], Radoslaw Bombera [1,§], Teresa Lettieri [1], Anne-Sophie Lequarré [2], Pascal Colpo [1] and Andrea Valsesia [1,*]

[1] European Commission, Joint Research Centre (JRC), Ispra, Italy; Rita.LA-SPINA@ec.europa.eu (R.L.S.); diana_conduto@hotmail.com (D.C.A.); radoslaw.bombera@gmail.com (R.B.); Teresa.LETTIERI@ec.europa.eu (T.L.); pascal.colpo@ec.europa.eu (P.C.)
[2] European Commission, Joint Research Centre (JRC), Brussels, Belgium; Anne-Sophie.LEQUARRE@ec.europa.eu
* Correspondence: andrea.valsesia@ec.europa.eu; Tel.: +39-0332789704
† These authors contributed equally.
‡ Current address: ECHA (European Chemicals Agency), Telakkakatu 6, 00150 Helsinki, Finland.
§ Current address: BioNavis LTD, Hermiankatu 6 8 H, 33720 Tampere, Finland.

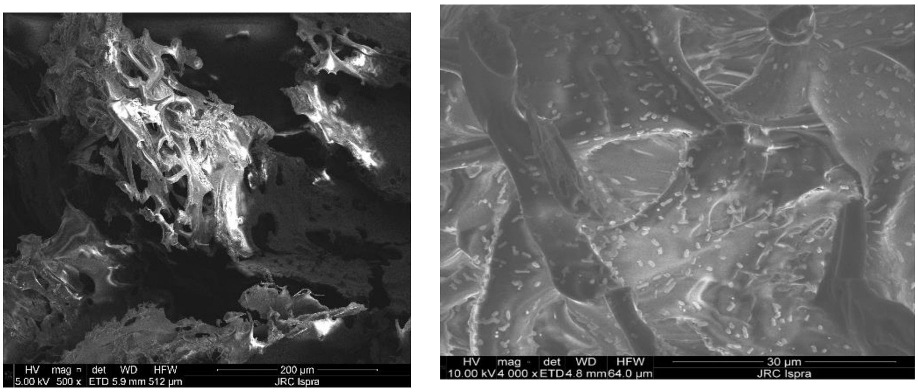

(**a**) (**b**)

Figure S1. Scanning electron microscopy image of (**a**) microporous cryogel (**b**) of *E. coli* bacteria adsorbed on the P(HEMA-AEM) cryogel.

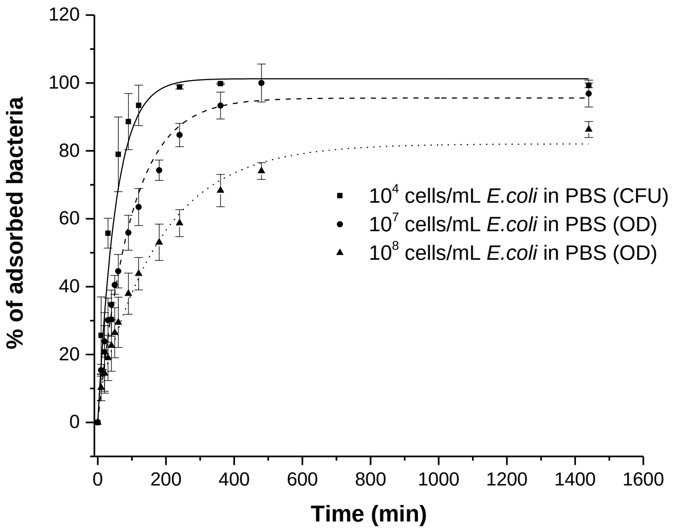

Figure S2. Adsorption kinetics of *E. coli* bacteria (initial concentration = 10^4, 10^7 and 10^8 cells mL^{-1}) onto P(HEMA-AEM) cryogel in PBS. The amount of adsorbed bacteria was determined by subtracting the concentration remaining in supernatant solution measured by turbidimetry (OD_{600nm}) and/or CFU quantification of the initial concentration. The ratio between the cryogel and the bacteria suspension was 45 mg of dried cryogel in 4 mL of PBS bacteria suspension.

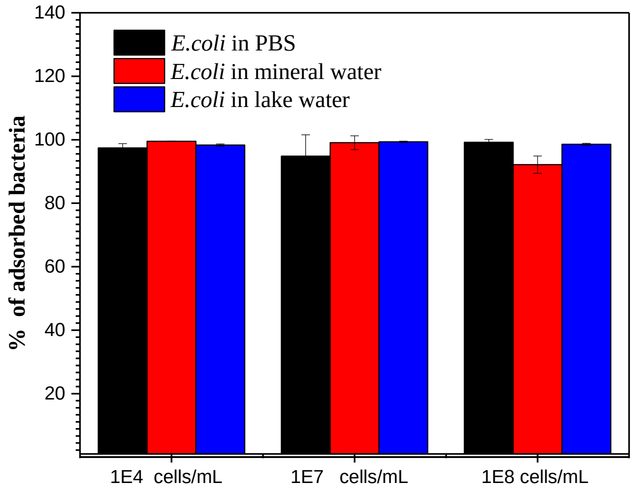

Figure S3. Comparative adsorption of *E. coli* bacteria at the concentration of 10^4, 10^6 and 10^8 cells mL^{-1} in PBS, in lake water and in commercial mineral water. 45 mg of dried cryogel were suspended in 4 mL of bacteria suspension.

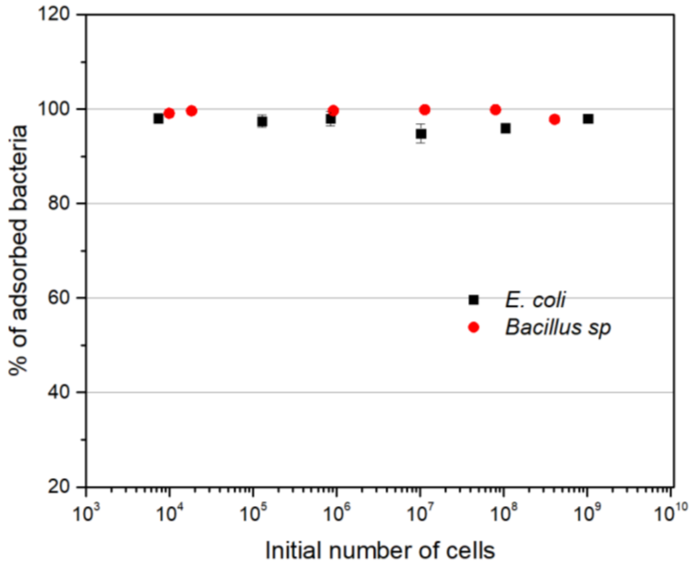

Figure S4. Adsorption of bacteria ranging from 10^3 to 10^8 cells mL^{-1} *E. coli* and *Bacillus sp*. bacteria keeping constant the ratio of cryogel and volume of bacteria to 45 mg per 4 mL.

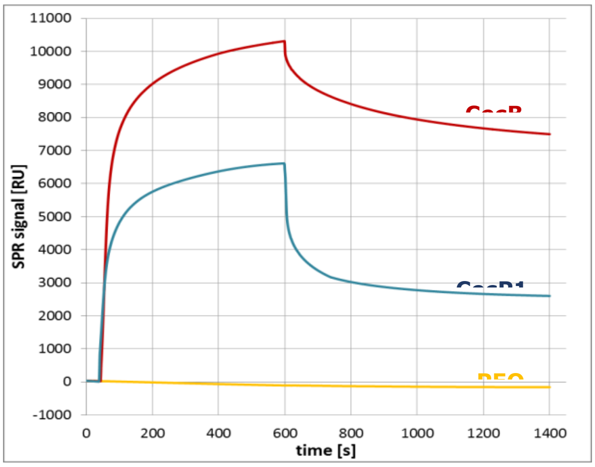

Figure S5. PEO Surface functionalization with Cecropin P1 (CecP1) and Cecropin B (CecB) monitored by SPR.

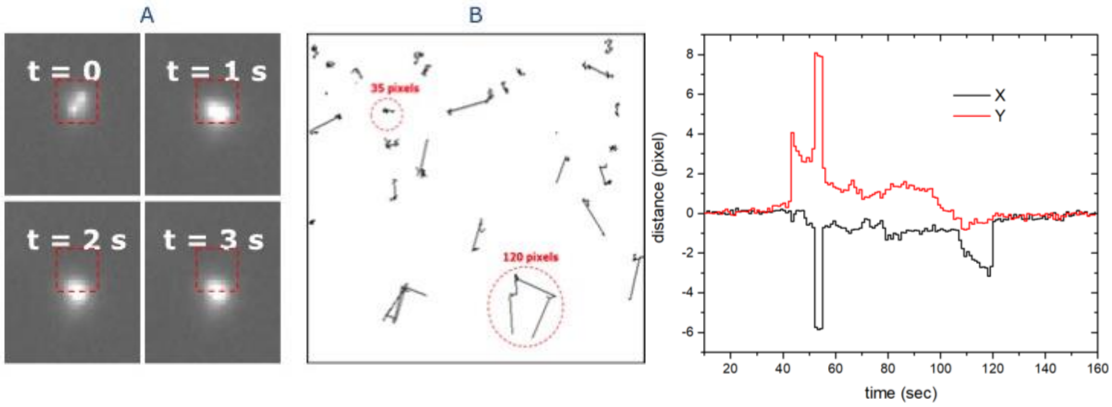

Figure S6. Cell motility is presented as (**a**) shifting of cell position regarding the geometrical centre at time zero, (**b**) vectorial travelled distance of the cell along the 160 frames and (**c**) variation of the X and Y position regarding the time zero (starting position).

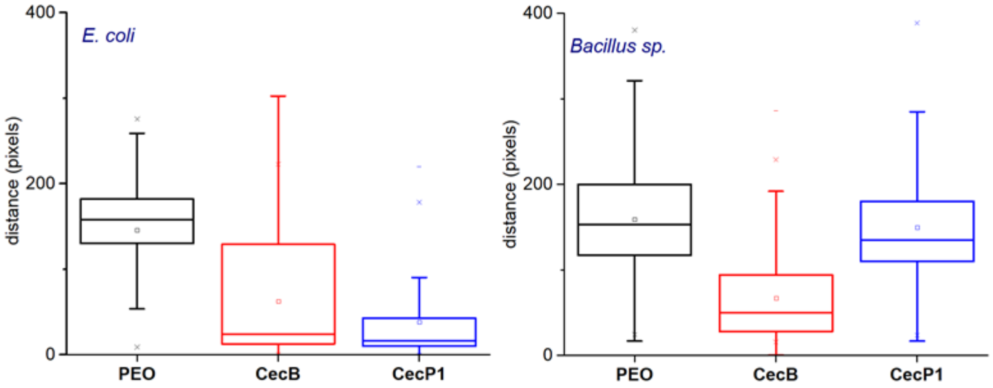

Figure S7. Motility analysis of (**a**) *E. coli* and (**b**) *Bacillus sp.* desorbed from the cryogel on PEO, Cecropin B (CecB) and Cecropin P1 (CecP1).
